# 

**Published:** 1855-10

**Authors:** 


### INDEX.

A.

PAGE.

American Dental Patents, Kendall on, . . . . . 36
Anomalous case of non-development of teeth, . . . 54
Address on Association, . . 62
Alimentary Canal, . . 155
Anatomy, Pathological, . . 159
Am. Jour. Science and Arts, . 160
Alumni Association of Dental Colleges, . . . 164—341
Animals, Sugar in the Liver of Hybernating, . . . . 165
Anæsthesia in Dental Surgery, 178
Adult Teeth, relative liability of, to Caries, . . . . 129
Air Chamber Plate, . . 242
Absence of all the Teeth, . 298
Anatomy, Manual of Human and Microscopical, . . 300
Amer. Med. Association, Transactions of, . . . . 338
Auditory Apparatus of Mosquito, . . . . . 343
Austen on Metallic Dies, . . 369
Abbey, Letter from . . . 404
Aldridge on Chloric and Hydrochloric Ether, . . . 449
Alveolar Abscess, Treatment, 458
Adhesive Plaster made of India Rubber, . . . . . 468
Alloying Gold, . . . 488
Allen and Hunter, . . . 492
Austen and Arthur, Correspondence between, . . . 496
Austen and Dubs, Correspondence between, . . . . 498
Anæsthesia by Congelation in Dental Surgery, . . . 505
American Dental Convention, 546
Arthur, a New Method of Using Gold Foil by, . . . . 600
American Soc. of Den. Surg., 666
Amalgam, . . . . . 666

B.

PAGE.

Badger, Dr. F. H., on Salivary Fistula, . . . . . 651
Block Work and Continuous Gums, . . . . . 56
Beale's Case, . . . . 76
Bate, C. Spence, on Destruction of Hard Palate, caused by Carious Teeth, . . . . 148
Bone, Excision of the Maxilla, 152
Bridges, M. K. . . . . 168
Butcher, R. G. H., on Extirpative of entire Upper Jaw, . 309
Bed-side, what to observe at, and after death, . . . . 337
Balt. College Dent. Surg., 340, 490
Ballard, C. W., on Treatment of Alveolar Abscess, . 458
Bones of the Head and Ear, Diseases of the, . . . . 465
Bases for Artificial Teeth, Gutta Percha, . . . . . 490
Brace of Worthies, . . . 667

C.

Chemistry of the Metals, 3, 169, 349
Cushman, C. T., on Anomalous Non-development of the Teeth, 54
Continuous Gum and Block Work, . . . . . 56
Crystalline Gold, Watts' Improved, . . . . . 123
Construction, Organization and Arrangement of Insane Hospitals, . . . . . 160
Curious Dent. Pathological Specimens, . . . . . 164
Caries of the Teeth, relative Liabilities of, . . . . 192
College of Dental Surgery, Baltimore, Valedictory to Graduates of, . . . . . 195
Causes of Dental Deformities, 223

PAGE.
Chambers' Atmospheric Plate, 242
Crystalline Gold, varieties, &c., of, . . . . . . 249
Continuous Gum Teeth, . 299
Coast Survey, Report on, . 340
College Dental Surgery, Balt., 340
Colleges, Alumni of American Dental, . . . . . 341
College of Dent. Surg., Phila., 343
Crystalline Gold, Illustrations of, 342
Crystalline Gold, . . 344
Copper, . . . . . 349
Caries of the Teeth, . . 392
Chloric and Hydrochloric Ether, 449
Chloride of Zinc, . . . 488
Curious Case, . . . 490
Contribution to the Museum of the Balt. Coll. Dent. Surgery, 495
Correspondence between Drs. P. H. Austen and R. Arthur, 496
Correspondence between Drs. P. H. Austen and C. H. Dubs, 498
Coffin, C. R., . . . . 503
Chemico-Physiologial Effects of Coffee, . . . . . 499
Crystal Gold and Gold Foil, . 531
Convention, American Dental, 546
Continuous Gums, . . 574
Crystalline Gold, . . . 590
Calvert, on Springing of Plates, 615
Chemical Analysis, Outlines of, 663
Clinical Medicine, Manual of, 662

D.

Dental Orthopœdia, . . 18

Dental Patents, . . . 36

Dental Obturators, . . 45

Dwinelle, W. H., on the Microscope, . . . . . 126

Dentition, Method of Directing Second, . . . . . 140

Dentistry, a Treatise on, . 161

Dental Deformities, causes of, 223

Dental Progress, Report on, 230

Dies, making Metallic, . 247

Dwinelle, on Crystalline Gold, 249

Dental Register, . . 343

Dental Knowledge, Importance of a mere thorough, . . 359

Dies, Metallic, . . . . 369

Dentition, Method of Directing Second, . . . . . 478

Dental Deformities, Causes of, 525

PAGE.
Degeneration in the Teeth, 605
Dental Associations, Proceedings of, . . . . . . 625
Digestion of Starch, . . 655
Diseases of Human Teeth, 664

E.

Ether, Physical Effects of, . 113
Excision of the Maxillary Bone, 152
Exodontosis of a Lateral Incisor with two Roots, . . 165
Extraction of Teeth, Hunt on the, 242
Extirpation of entire Upper Jaw, 309
Experimental Physiology, . 342
Ether and Chloroform, . . 381
Ether, . . . . . 449
Erichsen's New Mode of Operating on Hare-lip, . . 455
Extraction of Teeth, difficult cases of, . . . . . 490
Essay on Malignant Cholera, 663
Extraction of Teeth, Skillful, . 667

F.

Forms of the Skeleton and of the Teeth, . . . . . 159
Fowler's Treatise on Dentistry, 161
Facts for the People, relating to the Teeth, . . . . 161
Fermentation and Putrefaction, 409
Fatigue of the Voice, . . 451

G.

Gold, Direction for using Watts' Crystal, . . . . . 123
Gold, Crystal, . . . . 249
Gold, Crystalline, Illustratiens of, 342
Gold, Crystallized. . . 344
Gold, Sponge, . . 486, 489
Gold, Alloying, . . 488
Gutta Percha Bases, . . 490
Gastric Juice, . . . 501
Gold Foil, New Method of Using, . . . . . . 600
Gastric Juice, . . . . 653
Goitre, . . . . . . 656

H.

Helme, Mode for Operating upon loose or sensitive Teeth, 33

PAGE.
Harris, Address on the Advantages of Association, . 62
Hurts upon Extraction, . 242
Huxley on the Enamel and Dentine of Teeth, . . 333
Handy on Dental Knowledge, 359
Hemorrhagic Diathesis, . 487
Hunter and Allen, . . 492
Harvey, Dr., Three Cases from my Note Book, . . . 589

I.

Inhalation of Ether, . . 113
Improvements, . . . 166
Illustrations of Crystalline Gold, 342

J.

Jones, C. H., a Manual of Pathological Anatomy, . 159

K.

Kirkbridge, Hospitals for the Insane, . . . . . 160
Kilbourne, Dr. G. H., Obituary of, . . . . . . 504
Kölliker, Manual of Microscopical Anatomy, . . 400

L.

Lusus Naturæ, . . . . 54

Liver, Sugar in the, of Hybernating Animals, . . 165

Liver and Spleen, Relations of the, to Respiration, . . 166

Leslie on Dental Progress, . 230

Letter from Wm. R. Abbey, 404

Letter to Senior Editor, . . 344

Levison, Causes of Dental Deformities, . . . , 525

Leslie on Crystal Gold and Gold Foil, . . . . . . 531

Lock on Continuous Gums, 574

Letter to a Young Physician, 663

Lizard taken from the Throat, 667

M.

Mercury, . . . . 3, 169
Microscope, Essay on, . . 126
Method of Directing Second Dentition, . . 140, 478

PAGE.
Maxillary Bone, Excision of 152
Manual of Pathological Anatomy, . . . . . 159
Making Metallic Dies, 247, 369
Microscopical Anatomy, Manual of, . . . . . 300
Medical Association, Transactions of, . . . . . 338
Medical and Chirurgical Faculty of Md., Reports for 1854, 339
Mosquito, Auditory Apparatus of the, . . . . . 343
Mendl on Fatigue of the Voice, 451
Mineral Plate Bases, . . 489

N.

Non-development of Teeth, 54
Necrosis of the Lower Jaw, . 400
Neuralgia Facial, . . 587
Neuralgia, Case of Intermittent Cranial, . . . . 620

O.

Owen, Prof. R., Forms of the Skeleton and of the Teeth, 159
Ossification of the Pulp of a Tooth, . . . . . 165
Our Present Number, . 167
Operating on Hare-lip, . , 455

P.

Pathological Anatomy, . 159
Physicians' Visiting List for 1854, . . . . . . 160
Paralysis occasioned by the Extraction of a Tooth, . 167
Platina, . . . . . 245
Philadelphia Coll. Dent. Surg., 343
Porcelain Teeth, . . . 344
Piggot, A. S., Density of Sponge Gold, . . . . . 408
Pemberton on Fermentation and Putrefaction, . . . . 409
Professional Specimen, . . 469
Preparations of Cavities for Filling, . . . . . 470
Pathology of the Mouth, . 583
Pease on Degeneration in Teeth, 605
Plates, Springing of, . . . 615
Pancreatic Juice, . . . 654
Premature Dentition, . 665
Porcelain Teeth, . . . 666

Stunay

PAGE.

Q.

Quinton on Local Anæsthesia, 505

R.

Relations of the Liver and Spleen to Respiration, . 166

Robinson on Anæsthesia, . 178

Rehwinkel on Caries of the Teeth, . . . . . 392

Richardson, Case of Intermittent Cranial Neuralgia, . 620

S.

Salivary Fistula, case of, . 657
Stillé on Psycological Effects of Ether, . . . . . 113
Syme, Alimentary Canal, . 155
Sugar in the Liver of Hybernating Animals, . . . . 165
Scott, Dr., Obituary of, . . 168
Smull on Necrosis of the Lower Jaw, . . . . . 400
Sayles *vs.* Rumbles, . . . 469
Sponge Gold, . . 486, 489

PAGE.

Supernumerary Teeth. . . . 488
Shepherd, Dr., Obituary of, 503
Separation of the Teeth, . 648
Sugar in the Liver, . . 658
Supposed Injustice, . . 665

T.

Talma on Dental Orthopœdia, 18
Taylor on Second Dentition, 140, 478
Thompson, Facts for the People, 161
Talbert, Preparation of Cavities, 470
Thompson on Tic Douloureux, 587
Taft, Separation of the Teeth, 648
Tobacco Smoking, . . 656
Tooth Soap, . . . . 667

W.

Wright, Chemistry of the Metals, . . . . 3, 169, 349
Watts' Improved Crystal Gold, 123

Z.

Zinc, Chloride of, . . . 488

